# Developing Gram-negative bacteria for the secretion of heterologous proteins

**DOI:** 10.1186/s12934-018-1041-5

**Published:** 2018-12-20

**Authors:** Lisa Ann Burdette, Samuel Alexander Leach, Han Teng Wong, Danielle Tullman-Ercek

**Affiliations:** 1 Department of Chemical and Biomolecular Engineering, University of California-Berkeley, Berkeley, USA; 2 Department of Chemical and Biological Engineering, Northwestern University, Evanston, USA; 3 Department of Plant and Microbial Biology, University of California-Berkeley, Berkeley, USA; 4 Present Address: Department of Chemical and Biological Engineering, Northwestern University, Evanston, IL 60208 USA

**Keywords:** Protein secretion, Bacterial secretion systems, Recombinant protein

## Abstract

Gram-negative bacteria are attractive hosts for recombinant protein production because they are fast growing, easy to manipulate, and genetically stable in large cultures. However, the utility of these microbes would expand if they also could secrete the product at commercial scales. Secretion of biotechnologically relevant proteins into the extracellular medium increases product purity from cell culture, decreases downstream processing requirements, and reduces overall cost. Thus, researchers are devoting significant attention to engineering Gram-negative bacteria to secrete recombinant proteins to the extracellular medium. Secretion from these bacteria operates through highly specialized systems, which are able to translocate proteins from the cytosol to the extracellular medium in either one or two steps. Building on past successes, researchers continue to increase the secretion efficiency and titer through these systems in an effort to make them viable for industrial production. Efforts include modifying the secretion tags required for recombinant protein secretion, developing methods to screen or select rapidly for clones with higher titer or efficiency, and improving reliability and robustness of high titer secretion through genetic manipulations. An additional focus is the expression of secretion machineries from pathogenic bacteria in the “workhorse” of biotechnology, *Escherichia coli*, to reduce handling of pathogenic strains. This review will cover recent advances toward the development of high-expressing, high-secreting Gram-negative production strains.

## Background

The development of recombinant insulin and its production in *Escherichia coli* in the 1980s [1] launched an industry that comprises billion dollar markets, with products ranging from protein biologics ($91B) [2] to industrial enzymes ($4.8B) [3, 4]. In recent years, the chemical and structural diversity of protein products has expanded rapidly, with targets such as short anti-microbial peptides [5], antibody-like binding proteins for diagnostics [6], and protein biomaterials [7, 8]. These vast differences in physicochemical properties call for a variety of strategies to ensure that production of heterologous proteins is robust and scalable.

Secreting protein to the extracellular space increases initial purity and thus can decrease the complexity of downstream processing. Eukaryotic hosts such as *Saccharomyces cerevisiae*, *Pichia pastoris,* Chinese hamster ovary (CHO) cells, or HEK 293 cells secrete recombinant proteins through their native secretion machinery at high titers [9–12]. Mammalian cells, however, are slow growing, expensive to culture, have large batch to batch variations, and can be difficult to genetically engineer [10, 13]. Yeasts typically are faster growing but can suffer from genetic instability and clonal variation [14].

Bacterial hosts offer fast growth, relative genetic simplicity, and genetic stability [15]. Gram-positive bacteria such as *Bacillus subtilis* and *Streptomyces lividans* allow protein secretion directly to the extracellular medium through the general secretory pathway, as they lack an outer membrane (OM). Secretion stress response systems and folding chaperones provide extracellular quality control. *B. subtilis* has a high secretion capacity—titers on the order of grams per liter have been achieved [16–18]. Compatibility with heterologous proteins is largely limited to enzymes, however, and extracellular proteolytic degradation is a significant product quality issue [19]. *S. lividans* has low extracellular protease activity while retaining a high secretion capacity. Nevertheless, the range of compatible proteins remains narrow, and growth of filamentous organisms such as *S. lividans* is difficult at scale. Progress in engineering these and other Gram-positive hosts for protein production is well-summarized in several review articles [18–22].

Gram-negative bacteria, including *E. coli*, are favored as bacterial hosts for protein production—they can express a broad range of heterologous proteins at high titers and are robust in industrial-scale culture [23]. A typical production process involves intracellular product expression, which requires cell lysis and multi-step purification to isolate the product from the cellular contents [1, 2]. Though this process is used for many products at industrial scale—including insulin, interleukin-2 (IL-2), and human growth hormone (hGH)—intracellular expression introduces product and process development challenges [24]. For example, expression of IL-2 in *E. coli* required mutation of an internal cysteine to ensure proper disulfide bond formation after cell lysis [25]. Further, intracellular overexpression of recombinant proteins in bacteria often leads to the formation of insoluble aggregates, or inclusion bodies. Initial product purity is higher in inclusion bodies compared to soluble cytosolic expression, but product recovery requires solubilization and refolding steps that must be optimized for each product [26]. Finally, some classes of proteins, particularly biomaterials, are toxic when expressed intracellularly in Gram-negative hosts, which yields low titers [27, 28].

Gram-negative bacteria secrete few native proteins [29]. Thus, heterologous protein secretion from Gram-negative bacteria combines the advantages of bacterial production with broad substrate compatibility and the high baseline purity afforded by extracellular secretion. Gram-negative bacteria possess seven secretions systems—types I–VI and type VIII—that are known to secrete protein to the extracellular space [30]. Recombinant proteins are secreted successfully by five of these systems: type I, type II, type III, type V, and type VIII secretion systems (T1SS, T2SS, T3SS, T5SS, and T8SS, respectively) [24, 31]. Moreover, some proteins are natively secreted by *E. coli* via other, as-yet unknown mechanisms and are used as fusion partners to facilitate secretion of recombinant proteins [32–34]. As another alternative, the outer membrane can be engineered to release periplasmic protein [35, 36]. This review describes recent efforts to improve the capabilities of these secretion strategies to produce recombinant proteins at high titers. We outline characteristics that differentiate the suitability of the secretion systems as a tool for protein production, describe recent engineering successes, and discuss the future outlook for secretion system engineering in Gram-negative bacteria.

## Characteristics that differentiate secretion systems

Bacterial secretion systems can be divided into two classes, based on whether their cargo enters or bypasses the periplasm. One-step systems secrete proteins directly to the extracellular space from the cytosol, while two-step systems first export proteins to the periplasm through the general secretory (Sec) or twin arginine translocation (Tat) pathways. In this latter system, the target subsequently traverses the outer membrane to the extracellular medium. Table 1 provides a summary of each secretion system discussed in this review.

**Table 1:** Summary of Gram-negative bacterial secretion systems for heterologous protein production

Secretion system	Model organisms	Genetic locus	Model secretion tag	Master regulator
Type I	*Pseudomonas fluorescens*	*tliDEF*	TliA	
Type I	*Escherichia coli*	*hlyA, hlyB, hlyD*	HlyA	
Type II	*E. coli*	*gsp*	Unknown	
Generalized two-step	*E. coli*	N/A	YebF	
Type III	*Salmonella enterica*	SPI-1	SptP	HilA
Type III	*S. enterica*	*fli, flhDC*	FlgM (flagellar)	FlhD_4_C_2_ (flagellar)
Type III	Enteropathogenic *E. coli* (EPEC)	Locus of enterocyte effacement (LEE)	EspA	Ler
Type III	*Shigella flexneri*	*Shigella* virulence plasmid	OspB	VirB, VirF
Type III	*Yersinia enterocolitica*	pYV virulence plasmid	YopE	VirF
Type V	*E. coli*	*tps*	Pet	
Type VIII	*E. coli*	*csg*	CsgA	CsgD (not necessary for synthetic induction)

The type I and type III secretion systems are both one-step systems. In these pathways, protein cargo must be either completely or partially unfolded to secrete successfully. Still, several groups have shown that proteins exported by these systems can fold properly in the extracellular media, even when disulfide bridges or salt bridges are required [37–40]. In addition, one-step systems are not required for cell viability, which expands the engineering space available for these systems [41, 42].

Two-step systems, including the type II and type V secretion systems, provide access to useful machinery found in the periplasm, including foldases, chaperones, and other protein-folding enhancers [43, 44]. However, Sec is always essential for cellular viability, and Tat can be essential depending on the strain and growth conditions. Thus, using the Sec and Tat pathways can limit the engineering space of the system [31, 45].

**Fig. 1:**
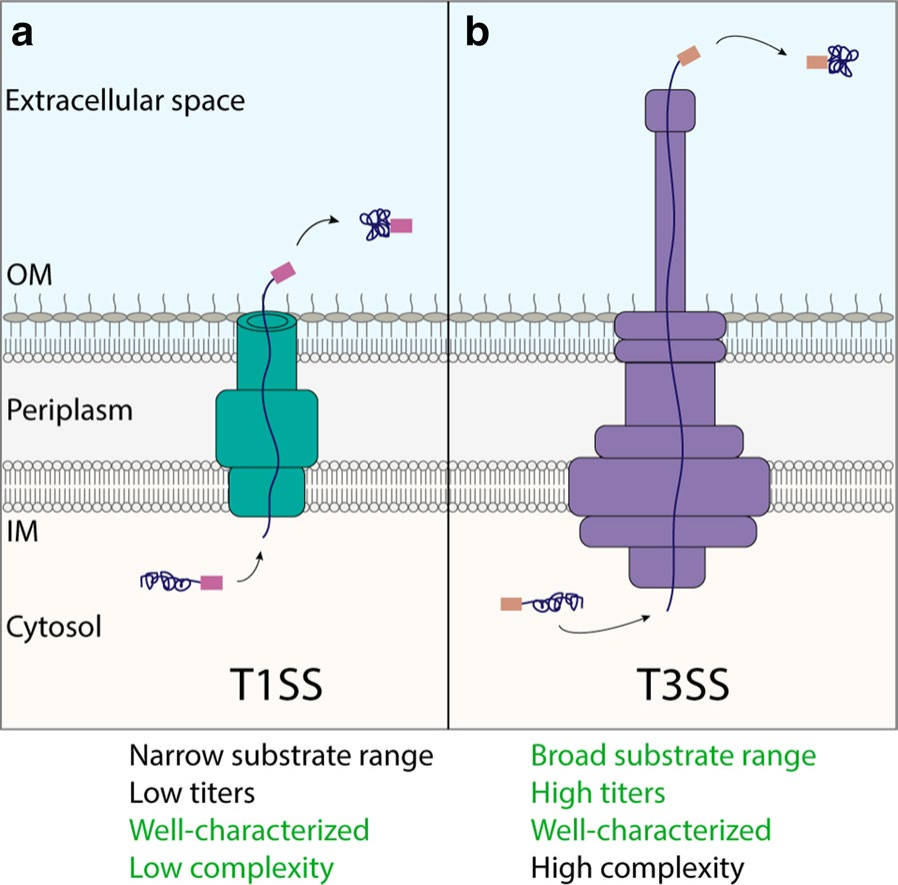
One-step secretion systems. Proteins (dark blue) are translocated directly from the cytosol to the extracellular space in an unfolded state, bypassing the inner and outer membranes (IM and OM, respectively). A C-terminal (T1SS, **a**) or an N-terminal (T3SS, **b**) secretion tag is required for translocation and remains attached to the cargo upon exiting the secretion apparatus. A list of engineering features for each secretion system is listed below the diagrams, and those highlighted in green are considered advantages of each system. “Substrate range” and the level of characterization refer specifically to heterologous protein secretion, and “complexity” describes the secretion machinery

**Fig. 2:**
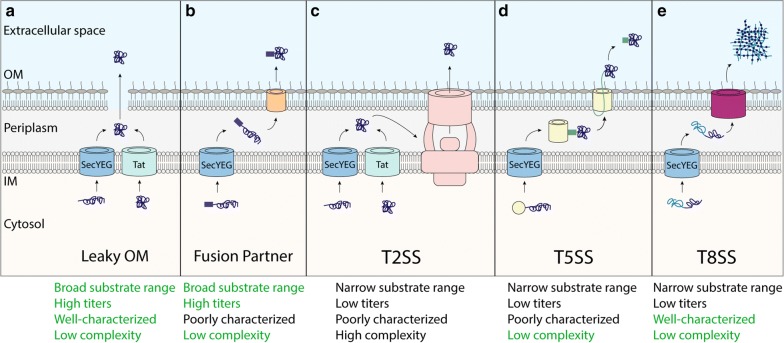
Two-step secretion systems. **a** Proteins (dark blue) are exported across the inner membrane (IM) via either Sec or Tat before passively diffusing into the extracellular space. **b** An example of transport via a fusion partner. Export pathway specificity is unknown for many fusion partners, but YebF (purple box) is secreted only when it is exported through Sec. It is believed to translocate the outer membrane (OM) via a porin (orange). **c** Proteins are exported through either Sec or Tat before entering the pseudopilus apparatus (pink) that transports cargo across the OM. **d** The translocation domain-passenger domain fusion is exported through the Sec pathway. The translocation domain (yellow) inserts in the outer membrane and the passenger domain (green) is secreted through the pore. An autocleavage event releases the passenger domain in the class of T5SS discussed here. **e** Proteins fused to the curli subunit (teal) are exported through Sec and are thought to traverse the outer membrane via an entropy gradient in a chaperonin-like structure (magenta). In the absence of the protein that anchors curli subunits to the outer membrane, fibers spontaneously polymerize and aggregate into networks in the extracellular space. A list of engineering features for each secretion system is listed below the diagrams, and those highlighted in green are considered advantages of each system. “Substrate range” and the level of characterization refer specifically to heterologous protein secretion, and “complexity” describes the secretion machinery

Before selecting a production platform, it is critical to understand the features of bacterial secretion systems that affect compatibility with a desired protein product (Table 2, Figs. 1, 2). While rigorous criteria have not been explicitly developed for any of the secretion systems discussed here, empirical evidence suggests that folding kinetics, folding complexity, and protein size can play a role in secretion efficiency [8, 37, 46]. Table 3 lists a selection of proteins that are successfully secreted by the systems discussed in this review.

**Table 2:** Features of Gram-negative bacterial secretion systems to consider when selecting a production platform

Secretion system	Number of steps	Secretion tag cleavage	Pathogenic origin	Access to folding chaperones	Secretion system induction scheme
Type I	One	No	Yes	No	Synthetic
Type II	Two	Unknown	Yes	Yes	Constitutive
Leaky membrane	Two	No tag needed	No	Yes	Constitutive
Unknown mechanism	Two	No	No	Yes	Constitutive
Type III	One	No	Yes	No	Synthetic
Type V	Two	Partial	Yes	Yes, but unfolded for transport across OM	Synthetic
Type VIII	Two	No	Yes	Yes	Synthetic

**Table 3:** A selection of heterologous proteins secreted by Gram-negative bacteria

Secretion system	Mechanism	Secretion product	Product type	Extracellular titer	References
Type I	HlyA	scFv	Antibody	2 mg/L	[68]
Type I	HlyA	CGTase	Industrial	0.58 mg/L	[124]
Type I	HlyA	Cutinase	Industrial	334 U/mL	[69]
Type I	HlyA	Interferon alpha 2	Therapeutic	6 mg/L	[37]
Type I	TliDEF, HlyA	Lipase	Industrial	8450 U/mL, 3 mg/L	[37,^61^]
Type I	TliDEF	Metalloprotease	Industrial	789 mg/L	[62]
Type I	TliDEF	Endo-β-1,4-mannanase	Industrial	4.65 mg/L	[75]
Type II and generalized two-step	TatExpress	hGH	Therapeutic	30 mg/L	[60]
	TatExpress	scFv	Antibody	N/A*	[60]
Type II and generalized two-step	Periplasmic expression via SRP	IgG	Antibody	236.5 mg/L	[96]
Type II and generalized two-step	Leaky OM	Human parathyroid hormone	Therapeutic	680 mg/L	[36]
Type II and generalized two-step	Leaky OM	Fab	Antibody	6 g/L	[125]
Type II and generalized two-step	Leaky OM	IFN-α	Therapeutic	N/A*	[95]
Type II and generalized two-step	Unknown, Cel-CD fusion	CGTase	Industrial	637.4 U/ml, 348 mg/L	[34, 104]
Type II and generalized two-step	Cel-CD fusion	Carbohydrate binding domain	Industrial	348 mg/L	[34]
Type II and generalized two-step	Cel-CD fusion	Neuritin	Therapeutic	211 mg/L	[34]
Type II and generalized two-step	YebF	α-amylase	Industrial	150 µmol glucose/min/mg protein	[54]
Type II and generalized two-step	YebF	Interleukin-2	Therapeutic	43,800 U/mL	[101]
Type II and generalized two-step	YebF	NanH2 Sialidase	Therapeutic	N/A*	[126]
Type II and generalized two-step	OsmY	Human leptin	Therapeutic	250 mg/L	[102]
Type II and generalized two-step	OsmY	Osteopontin	Therapeutic	3.6 mg/L	[100]
Type III	Flagellar	Interferon alpha 2	Therapeutic	0.6 mg/L	[50]
Type III	Flagellar	Lipase	Industrial	~420 U/L	[127]
Type III	Flagellar	Enfuvirtide	Therapeutic	13.4 mg/L	[128]
Type III	Flagellar	Apical membrane antigen 1	Therapeutic	N/A^*^	[89]
Type III	Flagellar	δ-SVIE	Therapeutic	N/A^*^	[89]
Type III	Flagellar	MrVIA	Therapeutic	N/A^*^	[89]
Type III	Flagellar	NCR peptide	Therapeutic	N/A^*^	[89]
Type III	Injectisome	Spider silk monomer	Material	14 mg/L	[27]
Type III	Injectisome	Elastin	Material	20 mg/L	[27]
Type III	Injectisome	Resilin	Material	20 mg/L	[27]
Type V	Type V	Pertactin	Therapeutic	1 mg/L	[39]
Type V	Type V	Ag85B	Therapeutic	N/A^*^	[39]
Type VIII	Type VIII	sdAb	Antibody	N/A^*^	[114]
Type VIII	Type VIII	Cecropin A	Therapeutic	294 mg/L (after purification)	[115]
Type VIII	Type VIII	Mussel foot protein	Material	N/A^*^	[117]

*The study did not report secretion titer

## Overview of engineering strategies

In addition to dividing bacterial secretion systems into two classes, the engineering strategies detailed in this review can be divided into four categories: (1) modifying the secretion tag that targets a protein for secretion; (2) engineering the secretion system machinery; (3) transferring a secretion system to a more desirable production strain; and (4) manipulating the genetic regulation of the secretion system.

All secretion systems described here require a signal sequence, or a peptide secretion tag that is appended to the N- or C-terminus of the protein cargo to facilitate secretion by the desired system [47]. Much engineering effort on the Sec and Tat pathways has uncovered clear design rules and minimal signal sequences that facilitate secretion through these systems. These rules form the basis of prediction algorithms that determine whether a given sequence is likely to be exported via one of these systems [47–49]. Similar approaches have been applied to other bacterial secretion systems, and the results suggest that secretion efficiency is highly dependent on the heterologous protein, though concrete design rules remain elusive [50]. Continuing this work to define design rules for secretion signal sequences will expedite process optimization for new protein products.

Altering the secretion apparatus can lead to higher secretion titer by improving the properties or increasing the capacity of the secretion system, though this approach is more feasible for secretion systems that are not essential to growth or survival [51, 52]. Traditional protein engineering tools, such as random mutagenesis, have been used in the directed evolution of the secretion apparatus with some success [50, 53]. However, limited sequence space can be surveyed using this approach. For future efforts, the development of high throughput screens and selections is critical to increase the utility of this strategy [54–56].

The ability to synthetically overexpress the secretion machinery is an important tool to maximize secretion titer [57]. This is especially the case for secretion systems that are not essential for growth and thus not constitutively expressed or highly expressed, such as the Tat pathway and the type III secretion system [41, 58–60]. Successes in this area include overexpressing master regulators that control the expression of the system [41, 58, 59] and synthetic expression of individual system components [60, 61]. This strategy increases the pool of secretion system machinery available for heterologous protein secretion.

Finally, many bacterial secretion systems are natively part of pathogenesis, and yet industrial-scale production requires safe and, ideally, non-pathogenic strains. Two approaches are being studied for using these systems in a non-pathogenic context. One strategy is to move the secretion machinery into a strain of *E. coli* that is already optimized for industrial protein production [58, 61–64]. Another viable strategy is to derive a non-pathogenic production strain from the pathogenic parent strain through genomic engineering [65, 66].

## One-step systems

### T1ss

#### System structure and overview

T1SSs transport a diverse array of proteins, from large pore-forming toxins to small hemophores [24, 67]. The T1SS is relatively simple: it requires only three components that are easily transferred to and overexpressed from a plasmid (Fig. 1a). The C-terminal signal sequences required for secretion via the T1SS contain repeats of a glycine-rich motif (G-G-X-G-X-D), often referred to as repeats-in-toxin (RTX) repeats as they were discovered in the RTX family of T1SS substrates [67]. Proteins are secreted in an unfolded state directly from the cytosol to the extracellular space, and the secretion signal sequence is not cleaved after secretion. Heterologous proteins such as cutinase, lipase, interleukin-6, and single chain variable fragments (scFvs) all are secreted successfully via the T1SS [24, 37, 61, 68, 69]. An anti-transmissible gastroenteritis virus scFv has the highest reported titer at 1–2 mg/L, though most reported titers are on the order of µg/L [68].

The primary drawbacks of using the T1SS for protein production are low titers, pathogenic hosts, and a limited heterologous substrate range [46, 70, 71]. Two systems, the HlyA system of pathogenic *E. coli* and the TliDEF system of *Pseudomonas fluorescens*, are the primary engineering targets for biotechnological applications. The TliDEF system poses the additional challenge of having limited secretion activity at temperatures that are optimal for cell growth [51]. Researchers have focused on increasing titer and expanding the range of compatible proteins in these systems by studying signal sequence composition, defining characteristics of well-secreted proteins, engineering the T1SS structure, and transferring the T1SS to lab strains of *E. coli*.

#### Signal sequence engineering

Minimal signal sequences were defined for both the TliDEF and HlyA systems by fusing truncations of the native substrates to the C-terminus of a heterologous protein and identifying the construct with the highest titer. In both cases, the number of RTX repeats determined secretion efficiency, or the percent of protein secreted compared to protein expressed, for several proteins tested. The final 60 amino acids of HlyA were necessary and sufficient for secretion, though increasing the length (and therefore the number of RTX units) increased secretion efficiency for some heterologous proteins [72, 73]. Proteins were secreted most efficiently by TliDEF when fused directly to the four RTX units present in its native substrate, TliA. Shorter and longer truncations of TliA decreased secretion titer for multiple heterologous proteins [74].

The RTX motif common to native T1SS substrates often provides compatibility with non-native systems. For example, the full-length *Serratia proteamaculans* metalloprotease, natively secreted by an *S. proteamaculans* T1SS, was secreted through TliDEF without adding a TliA secretion signal [62]. Testing signal sequences from different organisms can improve secretion efficiency, as observed when Eom et al. attempted to secrete endo-β-1,4-mannanase of *B. subtilis* with the TliDEF system. A TliA fusion caused undesirable leakage of cytosolic proteins, while a fusion to a truncation of PrtA, a T1SS metalloprotease from *Serratia marcescens,* produced active mannanase with a specific activity (10,103 ± 49 U/mg) similar to wild-type (10,108 ± 35 U/mg) without cytosolic leakage [75].

#### Understanding heterologous protein features that impact secretion

Several studies related folding kinetics to “secretability” with the HlyA system. In one study, Bakkes et al. found that wild-type maltose-binding protein (MBP) was poorly secreted, while slow-folding mutants fused to the same signal sequence were secreted at much higher levels [46]. This observation was reinforced when Schwarz et al. performed a similar experiment on intestinal fatty acid binding protein, a fast-folding eukaryotic protein. Again, only a slow-folding mutant was secreted by the HlyA system [40]. Additionally, Lenders et al. found that eGFP fused to the 218 C-terminal amino acids of HlyA (HlyAc) was unable to be secreted by the T1SS and stalled the secretion of HlyAc, presumably because folded protein blocked the translocator [76].

Recently, Khosa et al. discovered that adding an A/U-rich sequence, normally located upstream of the translation initiation codon in full-length HlyA, to the 5′ untranslated region of HlyAc facilitated secretion titers on the order of milligrams per liter for several heterologous proteins [37]. Secretion titer varied directly with increased transcript levels for all proteins tested, including wild-type MBP. The efficient secretion of wild-type MBP in the presence of the enhancer sequence led the researchers to suggest that the A/U-rich enhancer sequence may alleviate the requirement for slow folding kinetics. The researchers also noted that A/U-rich sequences are known to enhance protein expression by increasing translation efficiency [77], but they did not conduct additional mechanistic studies.

Another study showed that the compatibility of heterologous proteins with the TliDEF system depends on overall charge and isoelectric point (pI) [78]. From a panel of heterologous proteins with varied characteristics, only negatively charged proteins secreted. Attaching oligo-aspartate to lower the pI of negatively charged proteins increased secretion, while attaching oligo-arginine to increase the pI prevented secretion of negatively charged proteins. Structural modeling showed that TliD dimers form a positively charged pore at the substrate entrance site, which might explain this effect.

#### Engineering secretion machinery proteins

Random mutagenesis of the HlyA system ATP-binding cassette transporter and membrane fusion protein yielded mutants that showed increased secretion titer. However, secretion behavior varied with both temperature and heterologous protein. For example, one mutant provided an approximately tenfold increase in secretion titer for an scFv relative to wild type at 23 °C but a two-fold decrease in secretion titer relative to wild type at 37 °C. Sugamata et al. attributed the undesirable behavior at 37 °C to inhibited growth observed in the mutant upon IPTG induction. They observed a similar result for c-Myc-HlyA, but secretion of a tumor suppressor protein was unaffected by temperature [70].

The TliDEF system is also sensitive to temperature. Secretion titer sharply declines as temperature increases, with maximum titer observed at 20 °C and no secretion observed above 30 °C [51]. Lower temperatures slow cellular growth, which lengthens production time and therefore increases production costs. To overcome this challenge, Song and colleagues performed random mutagenesis on TliD and screened for mutants with TliA lipase function above 30 °C. They isolated a mutant that was secretion-active at temperatures up to 35 °C. Furthermore, the mutant strain secreted TliA at higher levels than wild-type TliD at all temperatures tested. Though secretion was observed at 35 °C, the system still favored lower temperatures—secretion at 20 °C in the mutant strain was tenfold higher than that observed at 35 °C.

#### Engineering non-pathogenic secretion strains

Two recent studies by Eom et al. demonstrated the strengths and limitations of exporting the secretion machinery from various pathogenic T1SS-containing strains to non-pathogenic, easier-to-handle *E. coli*. In the first study, *E. coli* were transformed with one each of four following T1SS systems: *P. fluorescens* TliDEF, *P. aeruginosa* AprDEF, *Erwinia chrysanthemi* PrtDEF, and *S. marcescens* LipBCD. The researchers attempted to secrete TliA through all four systems in *E. coli.* To their surprise, the highest titer of TliA was observed from secretion by LipBCD, even though it is not the native substrate of that system. However, in this study, the effect of differences in intracellular protein titer were not considered, including expression of the secretion machinery, the expression of TliA, or rates of intracellular proteolytic degradation [61]. In the second study, Eom et al. overexpressed TliDEF in both *E. coli* and its native host, *P. fluorescens*, and observed a 34-fold higher secretion titer of *S. proteamaculans* metalloprotease in *P. fluorescens* [62].

### T3ss

#### System structure and overview

There are two classes of type III secretion systems: the injectisome and the flagellar T3SS (fT3SS). Pathogens such as *Salmonella enterica*, *Yersinia enterocolitica*, and *Shigella flexneri* use the needle-like injectisome to secrete proteins that facilitate virulence into host cells. The fT3SS natively secretes and assembles the bacterial flagellum. Both T3SSs are multimeric protein structures that span the inner and outer membranes (Fig. 1b) [79]. Proper T3SS function requires additional regulatory proteins and sRNAs, so it is difficult to transfer the T3SS to a plasmid, much less a non-native host. An N-terminal secretion signal targets proteins to the secretion apparatus, where it is secreted in an unfolded state directly from the cytosol to the extracellular space. Some secretion signals require a cognate chaperone to carry out this function. The secretion signal remains attached to the secreted protein [80].

Researchers have successfully secreted high titers of a wide variety of proteins using these systems. Proteins such as scFvs, alkaline phosphatase, and spider silk monomers are secreted at titers up to 130 mg/L via the injectisome [8, 24, 38]. The flagellar T3SS secretes a variety of substrates as well, including GFP, neuroactive peptides, and α-enolase at titers up to 15 mg/L [81, 82]. The T3SS is particularly attractive for its ability to express and secrete otherwise difficult-to-express proteins [27].

Though researchers achieved high titers using the T3SS, design rules for compatible substrates and secretion tags remain elusive. Regulation of the T3SS is tightly controlled and sensitive to environmental input and growth phase [80]. Moreover, the T3SS injectisome in particular is found in pathogenic bacteria, which are undesirable hosts from an industrial perspective. Recent research has focused on characterizing the secretion signal, transferring the T3SS to non-pathogenic organisms, and overriding native control of the system to increase titer and decrease sensitivity to environmental input [38, 50, 58].

#### Injectisome

##### Strain engineering

Native T3SS injectisome machinery integrates a variety of input signals to ensure that T3SS machinery is constructed only when required for its pathogenic function. Although this strategy confers obvious evolutionary benefit, it often works counter to the goal of maximizing secretion titer. Thus, researchers have sought to manipulate and engineer regulatory pathways to exploit positive regulation and remove or circumvent negative regulation.

T3SS activation and assembly is controlled by at least one master regulator. Overexpressing a master regulator allows synthetic induction and can increase secretion activity in the population. In *S. enterica*, synthetic overexpression of the SPI-1 T3SS master regulator HilA increased the proportion of cells expressing the T3SS from around 30% to near 100% and increased secretion titer of a model protein tenfold [41]. This modification also promoted secretion of other heterologous proteins that were previously considered incompatible with the T3SS. Crucially, this increase in secretion titer and activity occurred in conditions that are known to repress the T3SS, such as high oxygenation, which is desirable to increase cell density and therefore bulk secretion titer.

Subsequent studies combined this work with strain engineering to secrete a variety of biopolymers at titers up to 130 mg/L [27, 52]. This titer was achieved by knocking out a protein present in the needle tip complex, SipD, and adding purified SipD exogenously. The *ΔsipD* strain increased titer 50-fold relative to wild type, and exogenous addition of purified SipD increased titer an additional twofold. The mechanism of the increase in secretion as a result of exogenous SipD addition has not yet been determined conclusively.

To remove all native regulatory inputs to the *S. enterica* T3SS, Song et al. recently reported a remarkable bottom-up approach to refactor the entire 35 kb SPI-1 locus, essentially rebuilding it from scratch [65]. The researchers removed non-essential genes, codon optimized certain genes, scrambled the gene order, and replaced non-coding genes with synthetic, controllable, well-studied genetic parts. The result was a minimal 16 kb SPI-1 that was about half the nucleotide length of wild type. The refactored system was completely controlled by synthetic regulation, and it was active in T3SS-repressing conditions. In addition to fully decoupling the T3SS from native regulation, this bottom-up approach revealed new information about necessary regulatory elements in the native system. For example, the researchers were able to identify at least two new essential factors—the small RNA, InvR, and an internal start site that generated a shorter version of the structural protein SpaO.

##### Engineering Non-Pathogenic Secretion Strains

The most popular approach for removing the T3SS from a pathogenic context is transferring the T3SS genes to a non-pathogenic host. This is a non-trivial endeavor because of its size and complexity, so a number of avenues have been explored. Much of this work is motivated by vaccine delivery efforts, as well-formed T3SSs can inject proteins directly into the cytosol of mammalian cells. The T3SS injectisomes of *Vibrio parahaemolyticus*, *Yersinia pestis*, and *S. enterica* can be expressed from plasmids in non-pathogenic *E. coli*; however, genomic integration of a synthetically controlled T3SS would create a more robust, programmable production strain [63, 83, 84]. One such strategy involved constructing a series of five rationally designed transcriptional units (TUs) that encoded the structural genes from the enteropathogenic *E. coli* (EPEC) locus of enterocyte effacement (LEE). Each TU was placed under the control of a synthetic Ptac promoter and integrated into a specific location in the *E. coli* K-12 genome to create a synthetic injector *E. coli* (SIEC) strain. The SIEC strain was capable of secreting native substrates expressed from a plasmid into HeLa cells [64].

Reeves et al. pursued yet another approach to transfer the *S. flexneri* T3SS into *E. coli* DH10B [58]. The researchers cloned the 31 kb of DNA necessary to express a functional T3SS from the *Shigella* virulence plasmid to a 44 kb synthetic plasmid that also contained the genetic elements necessary to transfer the T3SS to a defined “landing pad” on the DH10B chromosome. The resulting strain, mT3 *E. coli*, was secretion-active upon synthetic induction of a master regulator present on a separate plasmid. The strain was capable of secreting equal levels of endogenous proteins as native *S. flexneri* and successfully translocated proteins into HeLa cells. The researchers used this programmable, non-pathogenic strain to identify signal sequences that promoted secretion of therapeutically relevant proteins such as mammalian reprogramming factors and TALENs.

Creating a replication-deficient host can also abstract the T3SS from a pathogenic context, as demonstrated by Carleton et al. in *ΔminD S. enterica* [85]. The *ΔminD* strain divided aberrantly to produce minicells that lacked chromosomes and therefore could not replicate further. The minicells were metabolically active and could synthesize proteins from a plasmid introduced in the parent strain. The researchers created T3SS-active minicells by overexpressing the master regulator HilA and expressing the secretion target from a plasmid.

Ittig et al. engineered the host strain directly to reduce pathogenicity [66]. In this study, all of the native substrates of the *Y. enterocolitica* T3SS were knocked out, and a synthetic growth dependency on meso-2,6-diaminopimelic acid was engineered, making the strain consistent with biosafety level 1 standards. The engineered strain was capable of secreting a variety of heterologous proteins expressed from plasmids into HeLa cells.

##### High throughput screening methods

Some T3SS injectisomes, such as those of *S. flexneri* and *Y. enterocolitica*, are activated by the addition or depletion of small molecules [86, 87]. Lesser and colleagues recently showed that this property could be exploited to establish high-throughput screening methods for rapid engineering of the T3SS [56]. The *S. flexneri* T3SS is activated in the presence of the dye Congo red, so the researchers developed a solid plate-based assay in which liquid cultures were spotted onto solid media that contained Congo red and then transferred onto another solid plate that contained IPTG to induce the expression of the desired secretion product. A nitrocellulose membrane was attached to the plate that contained IPTG, incubated overnight, and probed with antibodies to detect secretion. This method allowed them to rapidly screen secretion titers of *S. flexneri* T3SS substrates in a multitude of conditions. The mT3 *E. coli* strain described previously was also compatible with this system. Though only a subset of T3SSs can be activated with a small molecule, the design of this screen could be adapted to other systems to enable rapid engineering.

#### Flagellar T3SS

##### Signal sequence engineering

The library of available flagellar secretion tags are empirically-determined truncations of native substrates [41, 59]. However, in two recent studies, researchers sought to characterize the secretion tags through a more systematic approach [50, 88]. In general, these studies revealed that secretion recognition and regulation via the fT3SS is complex, and that design rules might be challenging to define. For example, truncations of FlgB and FlgE fused to β-lactamase (Bla) required a native *flgB* 5′UTR for efficient secretion, while secretion of a FlgK-Bla fusion remained unchanged in the presence of the native *flgB* 5′UTR [88]. A long truncation of FliC fused to interferon-α (IFN-α) secreted better than a short truncation, while the inverse was true for MBP, and there was no difference in secretion titer for GFP [50]. Secretion efficiency depended strongly on the combination of the secretion tag and the heterologous protein, and no correlations have yet been identified.

##### Manipulating native regulation

The flagellar T3SS integrates numerous inputs in its complex regulatory circuit. Identifying critical nodes of control can increase secretion efficiency, as described above for the T3SS injectisome. A systematic evaluation of factors that could affect secretion titer via the *S. enterica* fT3SS determined that master regulator overexpression, increased chaperone stability, and high ionic strength (e.g. 200 mM NaCl) all increased secretion titer. Both knocking out native substrates and deleting SPI-1, which is known to participate in regulatory crosstalk with the fT3SS, had no effect or caused a slight decrease in secretion titer. However, combining the factors that improved secretion into an optimized strain facilitated secretion of difficult-to-express proteins such as conotoxins δ-SVIE and MrVIA, nodule-specific, cysteine-rich antimicrobial peptides (NCR), and a malaria surface antigen domain of apical membrane antigen AMA-1 [89].

##### Transferring the secretion machinery

The flagellar system is largely conserved throughout the domain of Eubacteria and thus improvements in one flagellar system can be applied to another. Many strains of *E. coli* currently used for biotechnological purposes lost their flagellar machinery during the domestication of those strains [90]. Auer and colleagues sought to restore flagellar machinery by inserting the fT3SS operon into the genome of the biotechnologically-relevant HMS174(DE3) strain and placing it under the control of the PlacUV5 promoter [59]. This construct was insufficient to generate observable FlgM secretion, but adding a second copy of the operon to increase gene products of the flagellar machinery and swapping the PlacUV5 promoter with the stronger T7 promoter produced measurable secretion titer. Absolute titer was not reported, making direct comparisons to the native fT3SS in *S. enterica* challenging.

## Two-step systems

### Sec and Tat

The Sec and Tat pathways are the common first steps for all two-step secretion mechanisms. These pathways, which are present in various domains of life, export proteins to the periplasm in Gram-negative bacteria. The Sec and Tat pathways are popular engineering targets for heterologous protein secretion, and these efforts are well summarized in other reviews [31, 91, 92].

One notable recent advancement is the creation of the TatExpress strain of *E. coli* [60]. This strain contains a genomically-integrated IPTG-inducible Ptac promoter upstream of the *tatABC* genes, which construct the machinery of the Tat system. By directly controlling the induction of the Tat machinery, researchers were able to export hGH and an scFv at higher titers than reported previously. They demonstrate this system is flexible to different heterologous proteins, stable, and can export at titers above 30 mg/L. Increasing export through Sec and Tat can increase secretion titer for any of the methods described below.

### Engineering a “leaky” outer membrane

Heterologous proteins are known to escape from the periplasm by various mechanisms [34, 39, 93], but some researchers work to increase outer-membrane leakiness as a production strategy. If a heterologous protein can be exported to the periplasm through Sec or Tat such that it comprises the vast majority of periplasmic protein, a leaky outer membrane should allow for efficient protein secretion without significantly increasing the complexity of the purification process. The major challenge with this strategy is ensuring that the membrane is not so weak that it spontaneously lyses, reducing cell growth. While secretion via periplasmic leakage is not a traditional two-step process, this strategy does rely on Sec and Tat pathways, and folded proteins are released into the extracellular space (Fig. 2a).

The integrity of the outer membrane can be modified environmentally or directly by knocking out genes involved in its composition. Though numerous environmental modifications are known to release proteins from the periplasm [35, 36], Wurm et al. sought to design a process compatible with protein expression in *E. coli* in large bioreactors. The optimal protocol was a post-culture incubation in 350 mM Tris for several hours followed by a mild heat shock at 38 °C. This process released 30% of periplasmic proteins with < 5% cell lysis [35]. In a subsequent study, the same group developed a simple method to monitor membrane leakiness by alkaline phosphatase activity and viability by flow cytometry and used that strategy to optimize culture conditions that maximized secretion titer [94].

Removal of bacterial lipoproteins and introducing defects in the peptidoglycan layer is a successful strategy for increasing outer-membrane permeability. In one study, a *ΔlppΔmrcB E. coli* strain was able to secrete human parathyroid hormone 1–84 coupled with thioredoxin as a fusion partner at titers of up to 680 mg/L [36]. A recent study by Gao et al. used transcriptomics to identify highly expressed lipoproteins that were likely to make the membrane leakier when deleted [95]. They measured secretion of IFN-α and polypeptides of varying sizes to evaluate size limitations. After performing iterative engineering on the single mutants, they identified two triple mutants, *ΔpalΔmrcAΔtolA* and *ΔpalΔmrcAΔompA*, that produced the highest bulk secretion titer for all proteins tested, including proteins up to 547 kDa, without a significant decrease in product purity relative to the single mutants.

#### High-throughput screening methods

Lee et al. developed a method to measure the amount of protein accumulated in the periplasm using the fluorescent arsenical hairpin binder (FlAsH) tag and periplasmic expression with cytometric screening, (PECS)-FlAsH [96]. This screen specifically measures export to the periplasm, though it is also a useful technique for improving protein secretion via two-step systems because export can be a bottleneck in protein production [97, 98]. The FlAsH tag is unable to penetrate the outer membrane in rich culture media [55], but the researchers found that resuspending cells in PBS was sufficient to allow the passage of the FlAsH reagent into the periplasm. Thus, after expressing a FlAsH-tagged heterologous protein and exporting it to the periplasm via the signal recognition particle (SRP) branch of the Sec pathway, cells were resuspended in PBS containing the FlAsH reagent to label the heterologous protein. The dye-protein complex was too large to escape the periplasm, so cells were screened using fluorescence-activated cell sorting (FACS). They used this method to identify a mutant from a transposon mutagenesis library that could export immunoglobulin G (IgG) and a G protein-coupled receptor at periplasmic titers up to 1.4 g/L in fed-batch culture.

### Fusion partners with unknown secretion mechanisms

Several proteins are found primarily in the extracellular medium but do not appear to be transported by any canonical secretion pathway [32, 34, 54, 99]. These include proteins natively secreted by lab strains of *E. coli* such as YebF and OsmY and heterologous proteins overexpressed in *E. coli* such as Cel-CD from *Bacillus* sp. Z-16 and α-toxin from *Staphylococcus aureus*. Though the full secretion mechanism is largely unknown for this class of targets, the four listed above are exported by a two-step mechanism that involves passage through the periplasm [32, 34, 54, 99] (Fig. 2b). These fusion partners, primarily YebF and OsmY, have successfully facilitated the secretion of numerous proteins including α-amylase, leptin, IL-2, and osteopontin [100, 101]. Though titers up to 700 mg/L have been reported [102], most proteins are secreted at titers on the order of 1 mg/L or less [34, 54, 99]. Thus, engineering efforts are focused on engineering signal sequences and establishing high-throughput screening methods to allow rapid identification and engineering of factors that affect secretion titer for these fusions.

#### Secretion tag engineering

Identifying the minimal amino acid sequence necessary for a fusion partner to guide a heterologous protein to the extracellular space minimizes the size of the fusion and might help elucidate the mechanism of secretion. By creating N-terminal truncations, Gao et al. revealed the minimum sequence necessary for secretion of the catalytic domain of a cellulose from *Bacillus* sp. Z-16, Cel-CD [34]. They created fusions to the minimal secretion sequence and to full-length Cel-CD and compared secretion titer in *E. coli* BL21(DE3) for several heterologous proteins. They observed a wide range of secretion behavior. Titers ranged from approximately 100 mg/L to 691 mg/L in shake flasks. The minimal secretion tag increased secretion titer for some targets and decreased secretion titer for others, but the authors did not thoroughly investigate the effect of expression or solubility of the fusions on secretion titer. In a subsequent study, Gao et al. were able to demonstrate that secretion of Cel-CD is Sec-dependent, though the mechanism of translocation from the periplasm to the extracellular space remains unknown [103].

Many heterologous proteins from *Bacillus* spp. are secreted to the extracellular space upon overexpression in *E. coli*, so evaluating the *Bacillus* secretome can yield new secretion signals [104]. Illias and colleagues computationally predicted signal sequences for 87 proteins highly secreted by *Bacillus lehensis*. Screening for compatibility with the Gram-negative Sec pathway using SignalP yielded 14 putative secretion signals. Fusions of these putative secretion tags to *Bacillus* sp. G1 cyclomaltodextrin glucanotransferase (CGTase) in place of its native secretion signal produced extracellular secretion when expressed in *E. coli* BL21(DE3). Unsurprisingly, they observed variable secretion behavior among the fusion partners.

Optimizing the Sec-specific secretion signal can also increase extracellular secretion titer. Domain swapping of the common Sec signal sequences DsbA and PelB produced a chimeric signal sequence capable of increasing extracellular secretion titer of *S. aureus* α-toxin H35L 3.5-fold [99]. For this protein, the composition and arrangement of amino acids in the hydrophobic core of the signal sequence strongly influenced secretion titer.

#### High-throughput screening methods

Because of variable, unpredictable secretion titer and the relatively undefined secretion mechanisms of these fusion partners, it is difficult to rationally design improvements to increase secretion titer. Thus, high-throughput screening is essential for expanding the development of these systems because it broadens the available engineering space. The primary target of these efforts has been YebF, a 10.8 kDa protein exported by the Sec pathway and subsequently translocated into the culture medium of lab strains of *E. coli* [54, 55], though these designs could be applied to any fusion partner.

Haitjema et al. screened an *E. coli* transposon library for strains that improved secretion of YebF using the FlAsH tag [55]. The FlAsH reagent was membrane-impermeable, so extracellular FlAsH-tagged YebF (YebF-FT) was detected without separating the extracellular medium from the cells. Eight gene deletions were identified that could increase YebF-FT secretion titer from 0.4 mg/L to as high as 13 mg/L. Secretion of cellulases from *Cellvibrio japonicus* also increased in the mutant strains, though the effect was variable among mutants. They demonstrated the universal applicability of the screening system by evaluating secretion with other fusion partners, the T2SS, and the T3SS.

The FlAsH tag-based screen is a powerful high-throughput tool, but it does require individual evaluation of each clone or library member. To allow examination of very large populations for mutants that increase secretion titer, Natarajan et al. developed a selection that couples protein secretion with cell viability [54]. The enzyme β-lactamase (Bla) provides antibiotic resistance to β-lactam antibiotics such as carbenicillin, and it is inhibited by binding to the 167-amino acid β-lactamase inhibitor protein (BLIP). Fusing BLIP to YebF establishes a selection for secretion-competent clones in the presence of carbenicillin. Poor secretors experience a growth defect in the presence of carbenicillin because YebF-BLIP is primarily localized in the periplasm and thus inhibits Bla, while clones that secrete YebF-BLIP at high titers to the extracellular space grew well on carbenicillin. This strategy allowed isolation of 13 mutants from the Keio knockout collection. Two of these mutants increased secretion titer of a YebF-cellulase fusion by more than 2.5-fold.

### T2ss

The T2SS spans the inner and outer membranes of Gram-negative bacteria. The Sec or Tat pathways export T2SS substrates to the periplasm, where the substrate enters the T2SS by an unknown mechanism and is secreted in a folded state into the extracellular space or the outer membrane [91] (Fig. 2c). T2SSs natively secrete a wide range of substrates in pathogenic and non-pathogenic bacteria, including toxins, cellulases, and c-type cytochromes [105]. The label “type II secretion system” has historically been applied to any extracellular secretion that passes through the Sec or Tat pathways, but as knowledge of bacterial secretion has expanded, the definition has become specific to the architecture described above [91].

Though T2SSs are present in numerous bacterial species, the mechanism of substrate recognition is unknown, and use of the system for protein production has been limited. Researchers successfully expressed the *E. chrysanthemi* T2SS from a plasmid in *E. coli* [106, 107], though they were only able to export its native substrates. Another study revealed that a cryptic, H-NS-silenced T2SS operon exists in *E. coli* K-12 that can secrete chitinase upon alleviation of H-NS silencing [108]. A significant impediment for future engineering of this system is the apparent strict species specificity of each T2SS [107].

### T5ss

Proteins processed through the T5SS are first exported to the periplasm through the Sec pathway. The T5SS is an autotransporter (AT)—a translocation domain inserts in the outer membrane, and the passenger domain is secreted through the pore created by the translocation domain without additional secretion signals or chaperones [109] (Fig. 2d). There are several subtypes of the T5SS that are defined by their oligomeric state and whether the passenger domain remains attached to the translocation domain or is cleaved after secretion [109]. Previously, the T5SS has been optimized as a cell surface display technology, primarily for antigen presentation for vaccine development [110, 111].

The T5SS has received less attention as a secretion platform than others discussed in this review. Nonetheless, several recent studies highlight the potential of this system. For example, researchers used a heterologously-expressed AT module from the *E. coli* protein, Pet, to secrete mCherry and antigens such as *Bordetella pertussis* Pertacin, *Y. pestis* YapA, and *Mycobacterium tuberculosis* ESAT6 at titers up to 5.4 mg/L in *E. coli* BL21 [39]. The Pet AT module is found natively in enteroaggregative *E. coli* and belongs to the serine protease ATs of the *Enterobacteriaceae* (SPATEs) class of T5SSs that release their passenger domains into the culture medium via an autocleavage event [39]. Henderson and colleagues determined the minimal signal sequence required to promote secretion using the Pet system and proved that another SPATE, the Pic system, could secrete folded and active heterologous proteins. However, they note that the system could be limited by complexity: protein containing disulfide bonds may not be secreted by the T5SS [112].

### T8ss

The T8SS, or the curli biogenesis pathway, is employed by *Enterobacteriaceae* to secrete amyloid fibers (curli) that constitute the major protein component of their biofilms. The T8SS, located in the outer membrane, is composed of three protein components that facilitate assembly of curli fibers in the extracellular space (Fig. 2E). Curli subunits are composed of a Sec signal sequence, a 22-amino acid T8SS secretion signal, and an amyloid core domain [113]. A periplasmic chaperone maintains the curli subunits in an unfolded state that prevents aggregation and facilitates secretion. Curli fibers are anchored to the outer membrane by a protein similar in composition to the curli subunit that is also secreted via the T8SS. Deletion of the anchor protein allows the fibers to spontaneously aggregate in solution.

T8SS engineering efforts have been conducted exclusively in *E. coli.* The *E. coli* T8SS can be overexpressed from a plasmid, but researchers have not compared secretion via a plasmid-borne T8SS to secretion by the genomic T8SS [114]. Active single domain antibodies (sdAbs), antimicrobial peptides, mussel foot proteins, and lanthanide binding domains are secreted as C-terminal fusions to the curli subunit CsgA [114–118]. Amyloid proteins from other organisms are secreted as C-terminal fusions to the combined Sec and T8SS signal sequences of CsgA [119], but that CsgA truncation has not been tested for secretion of non-amyloid heterologous proteins. Secretion via the T8SS might be limited by size—fusions of mCherry, Bla, and alkaline phosphatase to CsgA were not secreted by the T8SS [114].

In contrast to other bacterial secretion systems, T8SS engineering efforts have focused primarily on engineering the secreted curli fibers to make nanofibrous meshes with a range of properties. CsgA is secreted with C-terminal fusions of a wide range of peptide tags [120], which provides myriad options for functionalizing the amyloid fiber network, from enzyme scaffolding [121] to nanoparticle patterning [122]. This suggests the machinery is robust with respect to protein cargo identity, though the putative size limit remains an issue. Furthermore, Joshi and colleagues developed a simple method to purify genetically modified CsgA fibers via filtration with yields from tens to hundreds of milligrams per liter [123].

## Conclusions and future outlook

Recombinant DNA technology and protein production in model organisms enabled products that improve all facets of human life, from protein biologics for cancer treatment to enzymes for making cheese [3, 4]. Despite remarkable advancements in biotechnology over the past 40 years, many new protein products and those in development are challenging to produce in a cost-effective manner using the current suite of production organisms. Furthermore, the emerging concept of point-of-care, on-demand manufacturing for personalized medicine will require new production organisms and technologies because of the unique challenges of such a system [129, 130]. Finally, much of the cost of a new protein product is derived from its product development. Secretion systems in Gram-negative bacteria have the potential to address these needs.

Future efforts to develop secretion systems in Gram-negative bacteria for heterologous protein production will benefit from a more systematic approach to maximize secretion efficiency and titer. For example, defining design rules for secretion tag compositions, elucidating compatibility with desired heterologous proteins, and optimizing synthetic control schemes are all critical strategies for establishing a robust production platform. To achieve these goals, it is essential to develop high-throughput screening methods and selections such as those described for two-step secretion systems. While the FlAsH tag screen used to optimize secretion of YebF fusions is a promising strategy, other options are necessary for proteins or systems that are incompatible with that screen design.

In addition, optimizing a secretion system as a protein production platform requires adaptation to bioreactor culture and establishing a production strain that is generally regarded as safe (GRAS). To this end, the HlyA, TliDEF, SRP, and leaky *E. coli* systems all demonstrated increased secretion titer in batch or fed-batch bioreactor cultures [35, 61, 96, 124, 125, 131]. Furthermore, secretion of the protein product to the culture medium permits high-efficiency continuous culturing methods, which could dramatically increase titer [132–134]. Creating non-pathogenic, GRAS secretion strains broadens available applications—a GRAS strain is more amenable for use in contract manufacturing facilities and allows wide use in academic labs for protein expression.

Engineering efforts have focused primarily on increasing titer, but as titers approach industrially relevant levels, attention should shift to assessing product quality. Several groups measure enzyme activity as a proxy for secretion titer, but few analyze the impact of secretion on product quality. Secretion via the T3SS injectisome, for example, allowed isolation of full-length biomaterial proteins at > 90% purity, while cytosolic expression yielded truncations over a 30 kDa range, only 35–65% of which was the full-length desired product [27]. Understanding the impact of secretion on product quality will inform engineering strategies and improve production system selection.

Though bacteria remain limited in their ability to append post-translational modifications, many secretion systems facilitate proper disulfide bond formation. Compatibility with other post-translational modifications remains largely unexplored for bacterial secretion systems, but YebF in particular is secretion-competent in *E. coli* after biotinylation [135], disulfide bond formation [55, 101] and, excitingly, N-linked glycosylation [136].

Finally, while the remarkable diversity of bacterial secretion systems can in and of itself enable the production of a wide array of new proteins, additional secretion machineries exist in nature that have yet to be fully characterized. Both type IV and type VI secretion systems remain unexplored for their ability to secrete heterologous proteins.

Several of the engineered systems reviewed here are on the cusp of achieving industrially relevant titers of difficult-to-express proteins, such as spider silk monomers or antimicrobial peptides [27, 33] (Table 3). Bacterial secretion systems can also enable production of entirely new classes of protein products, as shown with the functionalized curli nanomaterials. In an industrial context, the short production times provided by bacterial secretion systems could decrease costs dramatically. Moreover, proteins secreted by these systems achieve an active form and often require only one chromatography step to isolate a pure product, further reducing costs [27, 37, 38, 40, 53, 69].

Using a bacterial secretion system to evaluate binding domains, biomaterial properties, or the efficacy of antimicrobial peptides would drastically decrease development timelines. Academic and industrial labs alike would benefit from inexpensive alternative production platforms for difficult-to-express proteins and new biotechnological targets. Shifting beyond the current paradigms of protein production could promote new technological innovation with higher purity, simpler systems, and lower cost.

## Abbreviations

AT: autotransporter
Bla: β-lactamase
BLIP: β-lactamase inhibitor protein
CHO: Chinese hamster ovary
EPEC: enteropathogenic *E. coli*
FACS: fluorescence-activated cell sorting
FlAsH: fluorescent arsenical hairpin binder
fT3SS: flagellar T3SS
GRAS: generally regarded as safe
hGH: human growth hormone
HlyAc: 218 C-terminal amino acid truncation of HlyA
IFN-α: interferon-α
IgG: immunoglobulin G
IL-2: interleukin-2
IPTG: isopropyl β-d-1-thiogalactopyranoside
LEE: locus of enterocyte effacement
MBP: maltose binding protein
OM: outer membrane
PECS: periplasmic expression with cytometric screening
RTX: repeats-in-toxin
scFv: single chain variable fragment
sdAb: single domain antibody
Sec: general secretory pathway
SIEC: synthetic injector *E. coli*
SPATE: serine protease ATs of the *Enterobacteriaceae*
SRP: signal recognition particle
Tat: twin arginine translocation pathway
T1SS: type I secretion system
T2SS: type II secretion system
T3SS: type III secretion system
T5SS: type V secretion system
T8SS: type VIII secretion system
YebF-FT: FlAsH-tagged YebF
